# School Readiness Test and Intelligence in Preschool as Predictors of Middle School Success: Result of an Eight-Year Longitudinal Study

**DOI:** 10.3390/jintelligence10030066

**Published:** 2022-09-12

**Authors:** Krisztián Józsa, Stephen Amukune, Gabriella Zentai, Karen Caplovitz Barrett

**Affiliations:** 1 Institute of Education, University of Szeged, 6722 Szeged, Hungary; 2 Institute of Education, Hungarian University of Agriculture and Life Sciences, 7400 Kaposvár, Hungary; 3 School of Education, Pwani University, 80108 Kilifi, Kenya; 4 Doctoral School of Education, University of Szeged, 6722 Szeged, Hungary; 5 Department of Human Development and Family Studies, Colorado State University, Fort Collins, CO 80523, USA

**Keywords:** preschool skills, intelligence, school achievement, middle school, longitudinal study

## Abstract

Research has shown that the development of cognitive and social skills in preschool predicts school readiness in kindergarten. However, most longitudinal studies are short-term, tracking children’s development only through the early elementary school years. This study aims to investigate the long-term impact of preschool predictors, intelligence, and mothers’ education on grade six school performance. This study presents the results of an eight-year-long longitudinal study. The sample includes 202 Hungarian children (89 boys) from a disadvantaged region of southeastern Hungary. The independent variables were the preschool measures: DIFER (Diagnostic System for Assessing Development), a widely used, standardized school readiness test that measures cognitive and social skills; the Raven intelligence test; and socioeconomic status. The dependent variables in grade six were: National Standardized tests in math and reading (NABC, National Assessment of Basic Competencies) and school grades (GPA). Cronbach’s alpha reliability of each test is above 0.76. Correlations and a series of multiple regressions were used for analysis. All three independent variables have significant predictive power for school performance in sixth grade. DIFER skills were the best predictors for reading achievement, intelligence for math achievement, and GPA was best predicted by mothers’ education. The results show that developing preschool skills, mothers’ education and IQ in preschool are essential to long-term learning success.

## 1. Introduction

School readiness has important implications for children’s school and life success, since it is associated with the development of academic achievement, self-regulation, peer relationships and communication skills (Fink et al. 2019). Children who start school with poor school readiness skills have challenges catching up with peers with higher levels of such skills (Burchinal et al. 2015; Duncan et al. 2015; Russo et al. 2019). Some scholars have also indicated that children who were not academically prepared upon school entry were more likely to suffer from social and personal problems such as unemployment, welfare dependence, dropping out of school, and criminal behavior in the following 10–25 year period (Burchinal et al. 2015). 

It is important to better understand the nature and sources of these long-term outcomes associated with school readiness. There is reason to believe that the long-term outcomes are mediated by continued poor school performance in the years following initial measures of school readiness (e.g., Davoudzadeh et al. 2015; Ricciardi et al. 2021). However, most studies examine only short-term effects, and those that have studied longer-term effects have only studied such effects through middle childhood/grade five. In some countries, the biological, psychological, and social changes associated with adolescence are accompanied by a change in school due to the transition from elementary to middle school at grade six, making it hard to understand the cause of the many changes that occur. However, in Hungary, the country in which the present research was conducted, grades six to eight are provided in the same school as earlier grades, enabling researchers to distinguish potential changes due to development from changes due to changes in schools and peer networks. Moreover, to the extent that continuities and discontinuities in school success are based on malleable, environment-sensitive characteristics, one would expect greater continuity in a continuous school setting. There is evidence of stability in changes in school readiness due to an intervention up to grade five (Welsh et al. 2020). But would such changes hold beyond middle childhood? To date, this question remains unanswered. 

There is evidence of an association between attendance at high-quality preschools and school readiness, particularly for children from lower socioeconomic status (SES) backgrounds (e.g., Akers et al. 2015; Amukune 2021; Rathbun and Zhang 2016). This evidence suggests that attending a quality early childhood program could contribute to reducing or ending intergenerational poverty for children coming from lower SES homes (Razza et al. 2015). Moreover, reducing the number of children with early learning difficulties before joining school contributes to decreasing the number of dropouts and improving school and life success (Pisani et al. 2018). Furthermore, econometric evidence suggests that return on investment is high if skill development intervention is strategically implemented at an early age compared to the adolescent stage, especially for children from disadvantaged backgrounds (Heckman and Mosso 2014). However, to date, research has not followed children into adolescence to ascertain whether early school readiness predicts academic success during adolescence, when behavioral health diagnoses, criminal behavior, and related outcomes are most likely to begin. Do the long-term academic difficulties originating in low school readiness continue beyond middle childhood, mediating the many long-term negative outcomes associated with school readiness, or are SES differences in other variables responsible for both difficulties in school readiness and these outcomes? In order to answer this question, it is first necessary to establish that early school readiness differences predict academic success during adolescence.

## 2. Theoretical Framework

### 2.1. School Readiness as a Predictor of Later Academic Performance

School readiness is a multi-dimensional concept that refers to competencies that facilitate school adjustment (Hair et al. 2006). Recent definitions of school readiness have emphasized not only the child’s need to be ready for school, but also the readiness of the school and community to accommodate the individual needs of the child, as indicated on school readiness assessments (Russo et al. 2019). Currently, there is no agreement among researchers on an operational definition of school readiness, although most scholars include, in some form, the five domains proposed by the National Education Goals Panel: approaches to learning; cognition and general knowledge; communication skills; health and physical development and emotional wellbeing (National Education Goals Panel 1991). The cognitive domain covers skills or knowledge of a particular subject, such as number or letter recognition or emerging literacy. Different theoretical models and assessments differ regarding the domain in which cognitive processing skills, executive functions, and/or approaches to learning are categorized. In some systems, these are categorized as cognitive skills (Diamond and Ling 2019), in some, they are considered a separate category called approaches to learning (Davis et al. 2021), and in other systems, they are considered part of socioemotional school readiness (Kälin and Roebers 2021). However, it is widely agreed that these are important areas of school readiness (Blair and Raver 2015; Sabol and Pianta 2017). Executive functions are general cognitive skills that facilitate planning, self-control, and learning (Willoughby and Hudson 2021). Recent neuroimaging findings have shown that the three main executive function skills, namely working memory, cognitive flexibility and inhibitory control, develop rapidly in early childhood and are crucial to school success (Sung and Wickrama 2018). 

Additionally, child language skills, including listening to and understanding a particular language (i.e., receptive language skills) and verbal communication with others (i.e., expressive language skills) are not only cognitive skills but also are instrumental in interactions with peers and teachers while at school. Another domain of school readiness is socioemotional skills, which encompass cooperation between peers, interactions with teachers, social relationships more generally, and the ability to self-regulate emotions, attention, and behavior. Children who can regulate their emotions and attention to academic tasks are better able to learn them, improving outcomes not only with reference to social relationships and behavior problems but also on academic tasks (e.g., Denham et al. 2012; Józsa and Barrett 2018). 

Several studies have reported the utility of school readiness domains at preschool in predicting GPA and standardized test scores such as math and language, up to grade five (e.g., Davoudzadeh et al. 2015; Ricciardi et al. 2021). Duncan et al.’s (2007) meta-analysis demonstrated that early math, reading, and attention skills, in that order, had the most significant power in predicting later academic achievement up to grade five. Moreover, socioemotional behaviors were non-significant predictors in this meta-analysis. However, more recent research has documented the importance of socioemotional skills as well (Ricciardi et al. 2021). Further, randomized controlled trials of social and emotional learning (SEL) interventions suggested that improvements in self-regulation seemed to mediate lasting improvements in cognitive skills (Jones et al. 2017). Duncan et al. (2020) used the Early Development Instrument (EDI), a teacher-report measure of cognitive, physical, and socioemotional school readiness, in kindergarten to predict third-grade mathematics and English language proficiency. EDI demonstrated strong predictive abilities in both subjects, even after controlling for neighborhood fixed effects and other child-level factors. 

Intervention strategies that target socioemotional and cognitive/academic school readiness have also improved academic performance later through the fifth grade. For example, Welsh et al. (2020) implemented a randomized controlled study of an intervention that adopted the Research-based, Developmentally Informed (REDI) curriculum, built on the PATH program for four-year-olds. PATH is a program that teachers deliver to promote socioemotional school readiness, including social problem-solving, emotional understanding, self-control and social skills, as well as language/emergent literacy (pre-reading) skills. Growth curve analysis demonstrated that intervention-based improvements in teacher-rated parent involvement, social adjustment, and academic engagement at Head Start were maintained from primary to fifth grades. 

Thus, research strongly indicates that school readiness is a good predictor of academic achievement through elementary school. Moreover, school readiness appears to be malleable. In a systematic literature review, Linder et al. (2013) identified seven factors reported in empirical research predicting school readiness in mathematics and literacy. These themes included (1) home environment, (2) learning-related skills, (3) child-care experience, (4) social behavior, (5) health and socioeconomic status, (6) mathematics and literacy-based tasks, and (7) family structure and parenting. This research suggests that most predictors of school readiness are appropriate targets for early intervention and that one of these malleable predictors is high-quality early childhood education. However, is school readiness predictive of academic success beyond elementary school? The importance of providing support for high-quality early education and parents of young children would be further underscored by research demonstrating that school readiness predicts even longer-term outcomes. Moreover, impact lasting until adolescence could help explain the association between early school readiness and impacts such as criminality and behavioral health difficulties. The present study is aimed at addressing the need to ascertain whether early school readiness is associated with academic success in early adolescence. 

### 2.2. Intelligence and Academic Performance

There is a strong association between intelligence and academic performance (Demetriou et al. 2020). Some studies report this association to range from 0.45 in elementary school and 0.54 in junior secondary, accounting about 29% of the variance of the academic grades (Roth et al. 2015). Józsa and Molnár (2013) used multiple regression to compare three predictors of academic achievement (cognitive persistence, Raven IQ scores, and a basic skills test) and discovered that cognitive persistence was the strongest predictor. In a related study, Józsa and Barrett (2018) explored the relationship between the affective aspects of mastery motivation and mathematics and reading scores at grade two, using structural equations modelling. Results indicated that negative reactions to challenge strongly predicted academic performance even after controlling for SES and IQ, suggesting that intelligence is not the only influence on academic performance. Demetriou et al. (2020) also reported that although fluid intelligence accounted for 39% of academic performance, 11% of this was an indirect effect of SES, and SES also had a direct effect that explained 4% of the variance, again suggesting that factors other than intelligence played a role in academic performance (Demetriou et al. 2020). In another similar study involving primary school children (third and fifth grade) and secondary school learners (seventh and ninth grade), fluid intelligence, personality, cognizance (self-evaluation of one’s cognitive abilities), and SES all contributed significantly to academic performance (Demetriou et al. 2019). Janurik and Józsa (2022) have found that IQ and early musical abilities in grade one together predict GPA in grade seven. Research on school readiness has also indicated an association between school readiness and intelligence (Pekdogan and Akgul 2016), suggesting the importance of determining how much school readiness contributes to academic performance after controlling for intelligence.

### 2.3. Socio-Economic Status, Intelligence and Academic Performance

Socio-economic status, defined by parents’ education, income and occupation, significantly predicts academic achievement and intelligence in childhood (SES; Dolean and Cãlugãr 2020; von Stumm et al. 2020). Such studies have reported that SES accounts between 5% (Bradley and Corwyn 2002) and 10% of the variance in school achievement (Sirin 2005), while intelligence explains up to 40% of academic achievement (von Stumm 2017). Some scholars argue that SES gives learners an advantage by providing a cultural context in which they learn the attitudes and work habits that are associated with school learning, regardless of their actual cognitive potential (Figlio et al. 2017; von Stumm 2017; Selzam et al. 2019). Similarly, quasi-experimental studies in which families’ income was experimentally raised led to an increase in academic performance in children from those families (Duncan and Magnuson 2011). Moreover, despite some scholars’ hypothesis that the contribution of intelligence to academic achievement is genetically based (Belsky et al. 2018), Hanscombe et al. (2012), studying children from low to higher SES aged two to eight years, found that SES moderated the environmental effect on intelligence but did not moderate the genetic effect on intelligence; children’s shared environments explained the higher variability of IQ in lower SES families (Hanscombe et al. 2012). 

As alluded to earlier, Demetriou and colleagues examined the relation between SES, cognitive ability and academic performance in preschool and secondary school (Demetriou et al. 2019; Demetriou et al. 2020). These two studies indicated that SES predicted school performance (about 15% of variance) and cognitive ability by a similar margin). 

However, importantly, SES also strongly predicts school readiness, suggesting that school readiness may be an unmeasured predictor that mediates the relation between SES and school performance. Thus, it is plausible to believe that both SES and intelligence are good predictors of academic achievement, although only a limited number of studies have empirically tested their predictive abilities from preschool to middle school. Apart from Demetriou et al. (2019) the majority of studies did not test the predictive ability of school readiness beyond five years. The contradictory results indicate that there is a need for further studies. Further, it is important to ascertain, between SES, school readiness, and intelligence, which variable contributes more to academic performance in a disadvantaged neighborhood. Therefore, in the present study, both SES and intelligence school readiness will be included as predictors, enabling assessment of the relative relation between each and school performance after controlling for the other.

### 2.4. The Education System in Hungary

The early childhood centre’s first stage of early education (infant/toddler school) provides professional daycare and development for children under three years of age. It is an optional service; only 18% of Hungarian children attend it (Józsa et al. 2018). The second early education stage is preschool, a state-funded institution offering education and care free of charge. Attending preschool is compulsory for all children from age three to six–seven; requests for exemption from compulsory attendance are only granted in duly justified cases. Preschools offer full-day (usually from 8:00 a.m. to 5:00 p.m.) care. BA is the minimum level of qualification for preschool teachers, whose assistant teachers help care for children. There are two preschool teachers per group. One works in the morning, the other in the afternoon, and they are both in attendance during lunch. The support staff are present for the whole day. The average group size is 22 children; by law, the maximum group size is 25 (Eurydice 2022; Golyán 2017). 

Children typically enter primary school at age six. Primary schools cover grades one to eight. Parents may request a one-year extension of preschool (kindergarten) attendance for six-year-old children. Primary and lower secondary education (ISCED 1–2) most often means the eighth-grade schools where children acquire all of their basic and intermediate skills. ISCED 1 covers grades one to four of primary school, while ISCED 2 covers grades five to eight. All students should attend ISCED 1 and 2 levels (Eurydice 2022). Participation in education is mandatory until age 16. 

There are three “tracks” of upper secondary education (ISCED 3): general secondary schools, vocational secondary schools, and vocational schools. General secondary schools provide general education, and typically prepare students for higher education. They cover grades nine to twelve, and students in this track take a secondary school qualifying exam at the end of grade twelve, a prerequisite for higher education admission. Vocational secondary schools are five years in duration, at the end of which students take a secondary school qualifying exam to earn a professional qualification and qualify for higher education admission. Again, this track provides general and vocational education and prepares students for higher education. Vocational schools last three years (grades nine to eleven). 

### 2.5. School Readiness Test Battery in Hungary

Early school readiness research in Hungary can be traced back to the 1970s (Nagy 1974a, 1974b). Then, under József Nagy’s leadership, research began to develop psychometrically robust tests to assess school readiness. During this period, empirical research on early childhood in Hungary began and strengthened progressively over time (Józsa 2016). In 1972, József Nagy and his colleagues developed a test system to assess school readiness and the intellectual, social, and physical development of children aged four to eight. Following small-sample pilot studies, a nationally representative study of 10,000 children was conducted in 1975. Such an extensive data survey in the research infrastructure of the 1970s was a huge accomplishment. The PREFER test (Preventive Development Assessment System for Children aged 4–7; Nagy 1976) was realized from this research. The book on standards for the PREFER test was published a few years later (Nagy 1980). At the beginning of the 2000s, the PREFER test system was revised and modernized. As a result of these developments, DIFER (Diagnostic Assessment Systems for Development between ages 4–8, Nagy et al. 2004a) was created. A decade later, computerized versions of some of DIFER’s subtests were also completed (Csapó et al. 2014).

DIFER is a widely used measure of school-related skills and school readiness in Hungary at present (Fleisz-Gyurcsik 2021; Gyurcsik 2020; Józsa et al. 2018). It helps teachers improve seven skills, each of which is a critical precondition to school-based learning. The seven skills included are pre-math, fine motor control, phoneme perception, understanding of cause and effect, deductive reasoning, the vocabulary of relations, and social skills. The test battery was standardized on a national representative sample including more than 23,000 children aged four–eight years (Nagy et al. 2004b).

The DIFER test battery is appropriate for determining school readiness and predicting academic success at the beginning of schooling (Józsa et al. 2018). It is not mandatory, but more than half of the preschools use it (Apró 2013; Gyurcsik et al. 2017). First-grade teachers administer the DIFER to one-third of their students (Educational Authority 2016). The teacher administers the DIFER tests if he/she thinks the child has some disadvantage or has less developed pre-academic skills and needs extra help. Then, based on DIFER’s diagnostic assessment, teachers set up an individual improvement plan for the child.

Skill improvement programs for preschool and elementary school children have also been developed concerning the areas measured in DIFER. A series of books has been written to help teachers implement improvement programs for ages four to eight. (Fazekasné Fenyvesi 2006; Józsa 2014; Józsa et al. 2017; Miskolcziné and Nagy 2006; Nagy 2009; Zsolnai 2006). There is research evidence that the DIFER improvement programs can effectively enhance disadvantaged children’s cognitive and social skills in preschool (Józsa 2016; Zentai and Józsa 2014).

Longitudinal studies have demonstrated that DIFER skills predict later school success. DIFER skills measured in preschool at the age of four years are moderately correlated with reading and mathematics at the end of first grade (Zentai and Józsa 2012). According to the results of another study, DIFER skills measured in the first grade are also moderately correlated with reading and mathematics performance in the fourth grade (Józsa and Csapó 2010). These results confirm that the DIFER test predicts later school success. However, these studies covered relatively short periods, lasting three years.

### 2.6. Hungarian National Assessment of Basic Competencies

Hungary has had a comprehensive National Assessment System since 2001, called the National Assessment of Basic Competencies (NABC). The content framework of the national competence survey was developed by integrating key aspects of existing international and domestic assessments, and the result of this integration was later enshrined into law (Act CXC of 2011 on National Public Education, https://net.jogtar.hu/jogszabaly?docid=a1100190.tv, accessed on 15 May 2022). The NABC aims to provide schools with objective, nationally comparable data on their students’ abilities in the two priority areas: reading comprehension and mathematics (Balázsi and Ostorics 2020).

The Hungarian Educational Authority (HEA) coordinates NABC measurements. All sixth-, eighth-, and tenth-grade students participate in reading and math assessments each year. The specific day of the measurement is prescribed by law at the beginning of each school year, and no other school activities can be organized for that day. Test booklets are centrally prepared and evaluated. The HEA publishes annual reports on the results. The reports inform the education administrators about the effectiveness of the education system. The HEA also advises the institutions’ administrators about the test scores for the institutions within their jurisdiction and informs the schools, parents and students about their testing outcomes.

The NABC framework defines the content and system of measurement. Test tasks are not based directly on the school curriculum but are measured by knowledge application to real problem situations (Szabó et al. 2018). The tasks of comprehension tests primarily measure the comprehension of written language used in everyday communication situations. Comprehensiveness and accuracy of the assessment are ensured, on the one hand, by including passages of different difficulty levels and, on the other hand, by the appropriate selection of comprehension tasks. Comprehension tasks include the thinking operations students perform after reading a written passage. The most straightforward operation is the search for explicit or implicit information. Recognizing the logical and content connections inherent in the text needed to find such information requires high-level thinking, with the highest level needed to interpret the passage as a whole (Balázsi et al. 2014).

Another area of competency measurement is mathematics, which is the application of mathematical knowledge learned in school to real-life situations. The test tasks can be divided into four content areas: (1) quantities, numbers, and operations; (2) functions and relationships; (3) shapes and orientation; and (4) statistics and probability. Within the mathematics test, the thinking operations required to solve the tasks, from simple, routine operations to the complex thinking required for complex problems, are also defined (Balázsi et al. 2014).

## 3. Research Questions

Despite other studies that have attempted to predict academic performance from intelligence, preschool skills and SES, we are unaware of any study that has predicted academic performance eight years later from all three of these predictors. We are also unaware of any study that has combined IQ, SES, and school readiness to predict grade six GPA and standardized reading and math tests. This is important, because grade six is often the transition to secondary education and marks the beginning of adolescence, when children are at greater risk for many serious negative outcomes. To fill this gap, the present study developed three research questions. First, what is the association of preschool skills with grade six GPA, math and reading? Second, what is the association of intelligence and mothers’ education with grade six GPA, math and reading? Third, what is the predictive power of preschool skills, mothers’ education, and intelligence on grade six GPA, math and reading? 

## 4. Methods

### 4.1. Sample

The data collection took place in six small settlements in Hungary. The primary consideration in selecting small settlements was that it was easy to follow the children there during the preschool-school transition. Additionally, these small towns are located in the disadvantaged region of southeastern Hungary. In these settlements, the proportion of the unemployed is higher than the national average, the parents’ levels of education are lower than the national average, and the proportion of disadvantaged children is higher than the national average.

We recruited 220 children aged 4–6 years (M = 4.59, SD = 0.32) who participated in the preschool study. Of these children, 202 were able to take assessments in the sixth grade. Unfortunately, 18 children had missing data due to relocation (N = 10) and/or illness (N = 8) and were omitted from all analyses. There were no significant differences between the retained and missing children’s SES. In the study, a total of 202 children were analyzed longitudinally, of whom 89 (44.1%) were boys.

### 4.2. Data Collection

The study is based on an eight-year longitudinal data collection. The first data collection point was at the beginning of the second year of preschool. The children had been in preschool for a year beforehand. At that time, we tested five DIFER skills: social skills, fine motor control, phoneme perception, the vocabulary of relations, and pre-math. The tests were administered by trained MA in Education students in two face-to-face sessions, taking an average of 15–20 min per session. Parents declared their highest level of education, from primary to university, on a demographic questionnaire. This was used as the measure of SES.

Eight years later, students took the Hungarian National Assessment of Basic Competencies (NABC) tests at the end of sixth grade. The math and reading tests were compiled nationwide by the Hungarian Educational Authority. All sixth-graders in the country completed these on the same day, under the same conditions. The assessments were administered by the staff of the Educational Authority using paper-and-pencil-based tests. The tests were completed, based on precise instructions, in the form of four school lessons, two in which the students solved the reading comprehension tasks, and two in which they completed the mathematics tasks. Each lesson lasted 45 min and was evaluated by the Educational Authority, from whom we obtained the data. Finally, we asked the children for their school grades.

Parents consented in writing to the processing of the data. In addition, the study’s ethical approval was obtained by the University Ethics Committee.

### 4.3. Measures

#### 4.3.1. Preschool Measures

##### Intelligence

We used Raven’s Progressive Matrices to measure non-verbal intelligence (Raven et al. 1998). The test comprises three sets of matrices, namely, A, AB, and B. The three sets are considered the three sub-scales of the test: A (11 items, Cronbach’s alpha: .89), AB (12 items, Cronbach’s alpha: .90), and B (12 items, Cronbach’s alpha: .88). The overall reliability for the 35 items was .89. The raw scores were transformed into percentage values ranging from 0 to 100.

##### DIFER

*Pre-math.* Pre-math includes a set of numerical counting (sequencing of positive integers in ascending and descending order), numerical manipulative counting (object operations), and number recognition. The test consists of five subtests, within which the items are ordered in ascending difficulty. The test consists of a total of 38 items.

*Phoneme perception*. The phoneme perception test is based on distinguishing speech sounds, and the child must perform sound differentiation between word pairs connected to color images. The test contains 15 items. 

*Vocabulary of relations*. This test measures words that express relations. Such relations include comparisons based on space, time, and quantity, for example, in front and behind, earlier and later, smaller or larger, longer or shorter, more or less. The test examines passive vocabulary and contains a total of 24 items. During the measurement, the part corresponding to the relational word must be shown to the child in pictures.

*Social skill.* Social skill is assessed based on triggering and monitoring children’s social activity during the tasks. Examiners use 1–5 point scales to rate children’s social skills. It contains 16 items. 

The *fine motor skills* test is recorded in groups of 4 children. The test sheet contained eight-line drawings. First, a visible drawing is provided from a given set of drawings for the children to copy. Then, after copying, the eight-line drawings are evaluated according to three criteria: content, placement and size. The fine motor skills test has 24 items.

##### Mother’s Education

The biological or adoptive mother, foster mother, stepmother, or primary female caregiver stated her own highest level of education. The highest educational attainment is classified into the following six categories, with the number of years spent studying in brackets: No primary school (<8 years of schooling), Primary school (8 years), Vocational training (11 years), Secondary school (12 years), BA degree (15 years), MA degree (17 years). The respective proportions of mothers in each category were: 2%, 21%, 33%, 33%, 10%, 1%.

#### 4.3.2. School Measures

##### Hungarian National Assessment of Basic Competencies (NABC)

*Math test.* The math test consisted of 56 tasks. Regarding the content areas, most of the tasks belonged to the topics of quantities, numbers, and operations, of which simple tasks required eight tasks, application and integrating operations required ten tasks, and only four tasks required the application of higher-order thinking operations, searching for and evaluating complex solutions. The content areas of probability and shapes and orientation received less emphasis than this, but the distribution of thinking operations developed similarly. The fewest tasks were in the field of statistics and probability calculations, which were only found in eight tasks in the test.

*Reading Test.* The reading comprehension test identified three types of text (experience, argument, informant), including 3-3 types of comprehension operations. Within the comprehension test, the proportions of both text types and comprehension action types were nearly identical. The comprehension test consisted of a total of 61 tasks, each of which was worth 1 point.

In the case of NABC tests, probability models are based on the Hungarian national sample. Tests use a two-parameter model in which each student is assigned a skill value, and each single-point item is assigned two parameters (Lak et al. 2015): the difficulty of the item (where the item is on the skill scale) and the slope (probability of solving the item), which increases with the ability of learners. For each task, the probability of success for each student can be calculated, a value between 0 and 1. This shows the development of the learner’s abilities and the parameters of the task. The development of mathematics and reading are standardized by a linear transformation calculated for each test task and then aggregated. This scale has an average of 1,500 ability points and a standard deviation of 200 ability points.

*Grade Point Average (GPA).* Students’ learning outcomes were recorded from school records. Students study 12 subjects, each rated 1–5 by teachers. The GPA was calculated from the 12 grades.

## 5. Results

### 5.1. Preliminary Analysis

Table 1 displays the DIFER subtests at preschool and the GPA, math and reading in grade six. The DIFER subtests were combined into one composite score (DIFER). The Cronbach’s alpha reliabilities of the tests and the mean and standard deviation of each test are also provided.

The Cronbach alpha reliabilities of all the subtests ranged from .76–.97, which is considered good reliability and suggests that all of these measures are reasonable to include in analyses of statistical relations with other variables (Gliner et al. 2017).

### 5.2. Intercorrelation of the Study Variables

The present study aimed to determine whether and how much various preschool school readiness skills predict academic achievement in grade six. Before determining to what extent the DIFER preschool skills (social skills, pre-math, phoneme perception, vocabulary of relations, and fine motor control), mothers’ education, and intelligence at preschool predicted academic achievement in grade six, we correlated the dependent and independent variables. 

Table 2 presents the intercorrelations of the independent variables at preschool. The most robust relations among specific school readiness skills were found between vocabulary of relations and social skills, which were correlated at the high moderate (*r* = .46, *p* < .01). All correlations, except those between phoneme perception and fine motor control, were significant. In addition to the significant correlations among most school readiness domains, DIFER at preschool was correlated at the high, moderate level with IQ, and all the DIFER skill domains were significantly correlated with IQ and mothers’ education. Note that significant correlations between DIFFER scales and the composite DIFER index are expectable, given that the composite is an aggregate of those scales. 

Table 3 displays the intercorrelations of the GPA, math, and reading tests in grade six; these are the dependent variables in the study. The GPA was developed from the 12 subjects that the students take, rated on a scale of 1–5 by the teachers. The math and reading test came directly from the students’ performance on the NABC standard tests. However, there were significant correlations with each other. GPA was strongly correlated with reading (*r* = .72, *p* < .01) and math tests (*r* = .66, *p* < .01) indicating they measured related constructs.

Table 4 shows the intercorrelation between the dependent variables (GPA, reading, math) and the independent variables (DIFER skills at preschool, intelligence and mothers’ education). Most interesting are the significant associations between the individual DIFER skills at preschool and the dependent variables at grade six. The DIFER subtests had moderate correlations with GPA, math, and reading, with the exception of phoneme perception, which had low but significant correlations with GPA (*r* = .24, *p* < .01), math (*r* = .25, *p* < .01) and reading (*r* = .24, *p* < .01). Across all the DIFER subtests, phoneme perception had the smallest correlation with all dependent variables. On the other hand, the correlations between IQ and the dependent variables ranged from moderate to strong (.45–.55), while the correlation of IQ with mothers’ education ranged from .37–.49. 

Among the independent variables, mothers’ education, DIFER and IQ were significantly associated with all dependent variables, with correlation coefficients ranging from moderate to high moderate (.37–.55). However, it is essential to ascertain how much DIFER skills, combined, contribute to predicting the outcome variables over and above IQ and mothers’ education. 

### 5.3. Regression Models

We developed three linear regression models to determine the ability of DIFER skills at preschool, IQ, and mothers’ education to predict GPA, math, and reading at grade six. Since all the dependent and independent variables demonstrated significant correlations, all the study variables were entered into the regression model. In case of the DIFER skills the five subtests were combined into the DIFER total score. 

Prior to conducting the regression models, we tested whether the statistical assumptions of this analysis were met. First, the sample size above 200 was sufficient given that only three independent variables were included in the analysis. Second, collinearity statistics, tolerance and variance inflation factor (VIF) values were examined (Table 5). All the tolerance values ranged from 0.76 to 1.0 and VIF between 1.0 to 1.34. The VIF values are smaller than 5, and all the tolerance values are greater than 1-R^2^, indicating that there were no multicollinearity concerns (Leech et al. 2015). Lastly, we checked the scatter and residual plots which all met the assumption of normality (Hair et al. 2019).

In the first regression model, the dependent variable was GPA, and DIFER, IQ and mothers’ education were independent variables. The second model had the same predictors, with the math test at grade six as the dependent variable, and the third model was reading (Table 5). 

#### 5.3.1. Regression Model One

We tested the explanatory power of the independent variables on grade six GPA. In this model, the dependent variable was GPA, and IQ, DIFER and mothers’ education were the independent variables. IQ, DIFER and mothers’ education were each significant predictors of grade six GPA (F (3, 198) = 42.671). Mothers’ education was the highest predictor with an explained power of 16% (rβ = .16, β = .33, t(3, 198) = 3.85, *p* <.001) while IQ (β = .25) and DIFER (β = .25) stood at 12% of explained power. The independent variables’ total explanatory power in predicting grade six GPA was thus 40%. 

#### 5.3.2. Regression Model Two

In the second multiple regression model, math was entered as the dependent variable, and again IQ, mothers’ education and DIFER were the independent variables (*F* (3, 198) = 41.561). In this model, IQ was the best predictor of math, with an explanatory power of 21% of grade six math (rβ = .21, β = .38, t(3, 198) = 5.90, *p* < .001) and mothers’ education the least, with an explanatory power of 6%. Mothers’ education, DIFER skills and IQ had total explanatory power of 30% in predicting grade six math. 

#### 5.3.3. Regression Model Three

In Model Three, reading was entered as the dependent variable, and IQ, mothers’ education and DIFER were retained as independent variables (F (3, 198) = 29.423). In this model the DIFER was the best predictor accounting for 12% of explained power (rβ *=* .12, β = .27, t(3, 198) = 3.89, *p* < .001) followed closely by IQ, with 11% explanatory power. Cumulatively, model three independent variables had an explanatory power of 30% in predicting grade six reading.

#### 5.3.4. Summary of the Regression Models

In summary, intelligence, mothers’ education and DIFER at preschool were strong predictors of GPA, math, and reading, with all three predictors contributing significantly to all three models. In the first model, which predicted GPA, the independent variables accounted for 40% (*R* = .63) of the variance, the same variables predicted 39% (*R* = .62) of the variance in math in the second model, and the same variables predicted 30% (*R* = .56) of variance in reading in the third model. According to Moore’s criteria, if *R*^2^ < 0.09, the predictive level is low, if 0.09 ≤ *R*^2^ < 0.49, prediction is moderate, and *R*^2^ ≥ 0.49 indicate high prediction (Moore et al. 2021). According to these criteria, all three models had moderate predictive abilities. Mothers’ education was the best predictor of GPA at grade six in Model 1 (β = .33), IQ was the highest predictor of math in model 2 (β = .38), and DIFER was the highest predictor for reading in model 3 (β = .27). However, in the three models, mothers’ education was the only predictor that accounted for less than 10% of the explained variance, which was true for both math and reading achievement.

## 6. Discussion

The present study aimed to determine long-term predictive abilities of intelligence, preschool school readiness skills, and mothers’ education at preschool on GPA, reading achievement, and math achievement. The study established that school readiness, mothers’ education and intelligence, all as assessed in preschool, all significantly predicted GPA, math achievement and reading achievement eight years later, in grade six, with each making significant contributions over and above the others. This is noteworthy, in establishing the importance of measuring not only IQ but also school readiness skills and SES, in order to better predict school success. Moreover, these three variables together were successful in predicting 30–40% of academic performance measures even after the important developmental change associated with adolescence had begun. 

Previous studies that have attempted to determine the ability of intelligence to predict academic achievement have mainly studied elementary children’s intelligence and future academic achievement (see Peng et al. 2019; Roth et al. 2015 for reviews). Such studies have also not combined preschool school readiness assessments with intelligence, and most used only academic performance scores rather than school grade measures such as GPA. GPA is an important measure to use because, unlike academic performance scores such as reading and mathematics achievement tests, which depend on an individual’s mental state at the single assessment time, GPA involves multiple assessments, often collected from multiple sources over long periods of time (Roth et al. 2015). In the present study, GPA was examined as an outcome measure, in addition to the standardized NABC achievement tests. We also examined scores on the NABC since the main objective of this examination is to assess the application of what students have learned in two key areas, mathematics and reading comprehension, and items do not come from the school curriculum, following international assessment trends (Balázsi et al. 2014; Balázsi and Ostorics 2020). Thus, a strength of the present study was its ability to examine the predictive power of DIFFER, IQ, and SES in relation to both a measure of students’ achievement in the Hungarian school system and in relation to international standards of achievement in math and reading.

The DIFER is able to assess seven skills, but for this study, we assessed the five skills which have been established to be the most reliable: social skills, pre-math, phoneme perception, the vocabulary of relations, and fine motor control (Nagy et al. 2004b). Apart from assessing school readiness, the DIFER is also helpful in assessing school-related skills and identifying children who might need extra support (Józsa et al. 2018). The vocabulary of relations and social skills were moderately correlated. Additionally, social skills were moderately correlated with math and reading. This agrees with other studies that recognize a link between social skills, self-regulation and academic performance (e.g., Claessens et al. 2009; Morgan et al. 2016). Further, the specific DIFER skills at preschool, except for phoneme perception, correlated moderately with GPA, math and reading at grade six. Contrary to our expectations, pre-math did not correlate especially highly with math; instead, they were correlated at a low to moderate but significant level that was slightly lower than the correlation of pre-math with reading. Intriguingly, both social skills and fine motor skills were more strongly correlated with math achievement. The primary objective of NABC is the application of math skills, while pre-math simply requires the learner to recognize numbers. Application of math skills requires much more complex thinking skills, reading, and math understanding (Hayati and Kamid 2019). It is clear that many skills, including fine motor skills that enable clear writing and social skills that enable children to seek help from peers and teachers, may play a role in math skill development. 

The vocabulary of relations had the strongest association with reading of any of the DIFER scales, with this correlation being stronger than the correlation between phoneme perception and reading (which also was significant). Phoneme perception is a necessary skill for reading, but unless one can understand the association between the sounds and the symbols, distinguishing visually “backwards” letters such as b versus d and upside down/backwards letters such as b versus p, one has difficulty learning to read. 

Fine motor skill had a moderate but highest association with GPA than any other DIFER skill, and also a higher correlation with GPA than the independent variables used in the regressions. The mode of administration of the fine motor skill assessment encourages the application of earlier learned skills and social skills, also captured in long-term assessment in GPA. Additionally, the association between GPA and reading achievement and math achievement was high, indicating they assessed related variables. However, GPA was developed by teachers from student grades and math and reading from standardized tests. This corroboration of outcomes from teachers and students confirms their mastery of math and reading. The correlation between intelligence and math achievement was more robust than the correlation between intelligence and reading achievement. This evidence is consistent with other studies. For example, our results are consistent with Peng and his colleagues’ meta-analysis of 680 studies that reported that intelligence is moderately related to reading and math, but the association was stronger for math than reading (Peng et al. 2019). It is also consistent with studies that established that intelligence is correlated with academic achievement, with a correlation ranging from .30–.70 (e.g., Colom and Flores-Mendoza 2007; Deary et al. 2007; Primi et al. 2010). A recent meta-analysis involving 240 studies also established that the correlation between intelligence and academic performance tends to be low in elementary school and moderate in senior high school. 

Finally, we operationalized SES as mothers’ education. Mothers’ education correlated moderately with GPA, math achievement and reading achievement. This result is congruent with other studies in the literature (e.g., Malecki and Demaray 2006; Sackett et al. 2009). Mothers’ education emerged as the best predictor of grade six GPA, although preschool school readiness skills and intelligence also contributed significantly to this model. Mothers with higher levels of education are likely to engage in practices in the home that enhance school readiness and school success, such as talking to children more, using more advanced vocabulary, and reading to children (Yamamoto and Imai-Matsumura 2019). Some scholars have gone a step further and investigated the role of genetic differences linked to SES, such as the cognitive abilities of the parents, in relation to their children’s school success (e.g., Belsky et al. 2018). However, such studies have reported contradictory results regarding the role of parental cognition in their children’s school achievement (Figlio et al. 2017). For example, Colom and his colleague in Brazil established that the role of the children’s own intelligence is the genuine predictor of academic achievement, since SES did not predict differences in scholastic achievement (Colom and Flores-Mendoza 2007). Although SES does not affect cognition directly, some studies have ascribed its relation to children’s school success to attitudes or habits related to learning that differ in families with SES disadvantage relative to those with SES advantage (Duncan and Magnuson 2011). Other scholars suggest that family environment, as associated with SES, enhances the realization of intelligence in reading or mathematics, especially at an early age (e.g., Peng et al. 2019). Moreover, it is possible that teachers are prone to allocate better grades to the children they perceive to come from families classified higher on SES. This tendency was observed anecdotally in the present study. 

In the second model, grade six math was best predicted by intelligence, then preschool school readiness skills and slightly by mothers’ education. Other studies using the Raven test to measure intelligence have found similar results. For example, Kyttälä and Lehto (2008) and Primi et al. (2010) found that intelligence predicted math better than reading. Some authors have also reported that empirically, intelligence predicts standardized achievement tests better- than measures of school grades, such as GPA (Deary et al. 2007). In the present study, GPA was predicted equally well by IQ and DIFER, with SES being the strongest predictor, highlighting the importance of including SES as a predictor. Moreover, in our third regression model, reading was predicted by the DIFER skills better than by intelligence, suggesting that IQ is not always the strongest predictor of standardized tests. Several longitudinal studies have, similarly, demonstrated the predictive abilities of school readiness tests (e.g., Duncan et al. 2020; Ricciardi et al. 2021; Welsh et al. 2020). In Hungary, other longitudinal studies adopting the DIFER have also exhibited the predictive power of the DIFER skills on academic performance, although they were for less than three years (e.g., Józsa and Csapó 2010; Zentai and Józsa 2012). 

## 7. Limitations

This study had several strengths; the longitudinal design ensured that the data collection was taken at several points from the same participants, which significantly improved the reliability and validity of the study, as well as our ability to infer that the preschool measures were truly predictive of the academic outcomes. Secondly, most of the data were collected directly from the students, and students’ actual teachers provided grades. These methods of data collection from authentic sources also enhanced the accuracy of the data. 

Despite the strengths, this study had some limitations. First, the data were collected from relatively small cities with mainly lower SES populations, both to represent this higher risk population and because this greatly facilitated follow-up of the families. However, this could limit the diversity of parents and children in our study. In particular, these settlements had many disadvantaged children from families with low SES, so it is not clear that results can be generalized to urban children with higher SES, even in Hungary. Third, although mothers’ education is arguably the most important aspect of SES, in terms of proximal influences of family SES on children’s achievement (Bradley and Corwyn 2002) it is only one aspect of SES, with other important indicators including parental occupational prestige and income, and, according to some researchers, social factors, such as family size, family structure, and residence area (Gorard and See 2009). 

Future studies should be done in which settlements with higher SES are included for comparison purposes, and a wider array of SES measures is included. Additionally, it would be useful to obtain parental reports regarding their children’s learning experiences at home to better understand potential mechanisms of SES.

## 8. Conclusions

The present study examined the predictive power of intelligence, preschool school readiness skills, and mothers’ education on later achievement in math, reading and GPA. The cognitive, social, and fine motor skills assessed by the DIFER in preschool were moderately associated with mothers’ education, intelligence and the dependent variables (math, reading and GPA). Mothers’ education, intelligence and preschool readiness skills significantly predicted grade six math, reading, and GPA. These preschool variables are individually and collectively essential predictors of sixth-grade school achievement, highlighting the importance of including all three, and, in particular, including the highly malleable school readiness measures. The best predictor for GPA was mothers’ education; the best predictor of math was intelligence, and preschool school readiness skills best predicted reading. However, all three predictors explained significant variance in all three outcome variables, despite the latter being obtained a full eight years later. These results confirm the utility of preschool school readiness skills, mothers’ education, and intelligence in predicting school success beyond elementary school. 

The importance of including highly malleable school readiness skills is that these can be modified by high-quality early childhood education, and school readiness assessment profiles can inform individualized remediation. The study thus highlights the potential importance of high quality early childhood education that is individualized to the needs of each student, particularly children from lower SES families. The results align with previous studies indicating that the development of basic preschool skills can greatly contribute to the success of later school learning and the proper development of math and reading skills. This study reiterates the need to focus on the development of preschool skills: social skills, fine motor control, vocabulary of relations, phoneme perception, and pre-math, all of which have an association with reading, math, and GPA in early childhood. Effective skill improvement programs are available in Hungary, programs which directly assist the work of preschool and elementary school teachers. The basis of the improvement programs is diagnostic tests in preschool practice. Diagnostic assessment supports individual education plans; Hungary has adopted the DIFER for ages four to eight, and this study supports the utility of DIFER for predicting long-term outcomes. Early childhood education programs are crucial for remediating any deficits in school readiness, particularly for disadvantaged children. 

## Figures and Tables

**Table 1:** Reliability and Descriptive Statistics.

Tests and Subtests	Number of Items	Cronbach’s α	Min–Max	Mean	SD
*Preschool*					
Social skills	24	.97	20–93	56.87	13.07
Fine motor control	24	.87	4–75	31.35	15.42
Phoneme perception	60	.93	12–100	80.17	15.09
Vocabulary of relations	24	.76	33–100	69.53	16.51
Pre-math	38	.90	9–81	41.71	14.83
DIFER	170	.97	22–80	55.93	9.95
*Grade 6*					
GPA	12	.91	1–5	3.97	0.70
Math test	67	.90	1079–2059	1436.39	175.87
Reading test	80	.92	971–1961	1428.76	184.92

Note: DIFER is a combined score of five subtests; GPA-Grade Point Average.

**Table 2:** Intercorrelations of the Independent Variables (Preschool).

Variables	1	2	3	4	5	6	7	8
1. Social skills	−							
2. Fine motor control	.27 **	−						
3. Phoneme perception	.32 **	.11	−					
4. Vocabulary of relations	.46 **	.22 **	.31 **	−				
5. Pre-math	.42 **	.21 **	.38 **	.35 **	−			
6. DIFER	.72 **	.55 **	.64 **	.72 **	.70 **	−		
7. IQ	.32 **	.40 **	.27 **	.29 **	.28 **	.47 **	−	
8. Mothers’ ed.	.24 **	.21 **	.18 *	.21 **	.26 **	.33 **	.32 **	−

Note: * significant at *p* < .05, ** correlations are significant at *p* < .01.

**Table 3:** Intercorrelations of the dependent Variables (Grade 6).

Variables	1	2
1. GPA	–	
2. Math test	.66 **	–
3. Reading test	.72 **	.70 **

Note: ** correlations are significant at *p* < .01.

**Table 4:** Correlations of the Independent and Dependent Variables.

Variables	GPA	Math Test	Reading Test
1. Social skills	.34 **	.37 **	.34 **
2. Fine motor control	.40 **	.36 **	.27 **
3. Phoneme perception	.24 **	.25 **	.24 **
4. Vocabulary of relations	.30 **	.32 **	.35 **
5. Pre-math	.29 **	.29 **	.30 **
6. DIFER	.47 **	.48 **	.45 **
7. IQ preschool	.47 **	.55 **	.44 **
8. Mothers’ ed.	.49 **	.37 **	.37 **

Note: ** correlations are significant at *p* < .01.

**Table 5:** Linear Regression Models.

Regression Models	Unstand. Coeff.		β	t	Sig.	r	rβ	Collinearity	
	*B*	*SE*						Tolerance	VIF
*1. GPA (grade 6)*									
Constant	1.75	.23		7.64	<.001				
DIFER Preschool	.02	.01	.25	3.90	<.001	.47	.12	.76	1.32
IQ preschool	.01	.01	.25	3.85	<.001	.47	.12	.88	1.14
Mothers’ ed.	.23	.04	.33	5.44	<.001	.49	.16	.79	1.26
*2. Math test (grade 6)*									
Constant	911.40	57.87		15.75	<.001				
DIFER Preschool	4.35	1.14	.25	3.82	<.001	.48	.12	.76	1.32
IQ preschool	4.35	.74	.38	5.90	<.001	.55	.21	.88	1.14
Mothers’ ed.	28.51	10.44	.16	2.73	<.001	.37	.06	.79	1.26
*3. Reading test (grade 6)*									
Constant	898.06	64.61		13.90	<.001				
DIFER Preschool	4.95	1.27	.27	3.89	<.001	.45	.12	.76	1.32
IQ preschool	3.07	.82	.26	3.73	<.001	.44	.11	.88	1.14
Mothers’ ed.	36.64	11.65	.20	3.14	<.001	.37	.07	.79	1.26

## Data Availability

Data are available upon request due to privacy restrictions.
